# Botulinum toxin injections as an effective treatment for patients with intertriginous Hailey-Hailey or Darier disease: an open-label 6-month pilot interventional study

**DOI:** 10.1186/s13023-021-01710-x

**Published:** 2021-02-18

**Authors:** Isabelle Dreyfus, Aude Maza, Lauriane Rodriguez, Margot Merlos, Hélène Texier, Vanessa Rousseau, Agnès Sommet, Juliette Mazereeuw-Hautier

**Affiliations:** 1 Reference Centre for Rare Skin Diseases, Dermatology Department (CRMRP), Larrey University Hospital, 24, Chemin de Pouvourville TSA 30030, 31059 Toulouse, France; 2 Department of Clinical Pharmacology, CIC1436, University Hospital, Toulouse, France; 3 Paul Sabatier University, Toulouse, France

**Keywords:** Botulinum toxin, Hailey hailey disease, Darier disease, Quality of life

## Abstract

**Background:**

Patients with Hailey-Hailey and Darier diseases present with disabling inflammatory lesions located in large skin folds, which are often exacerbated or induced by sweating. Quality of life is highly impaired because of pain and recurrent skin infections. An improvement in skin lesions after botulinum toxin A injections has previously been reported in some patients but no prospective interventional studies are available. The aim of this open-label, 6-month, interventional pilot study (NCT02782702) was to evaluate the effectiveness and safety of botulinum toxin A for patients with moderate to very severe skin lesions located in folds.

**Results:**

Thirty patients (26 Hailey-Hailey/4 Darier) were included. Botulinum toxin A proved effective within the first month in two-thirds of patients, taking all study parameters (itchiness, cutaneous pain, sweating and odour, infections, psychosocial impairment and quality of life) into account and persisted during the 6-month follow-up period. No patient was classed as a BtxA non-responder, but 11 (37%) Hailey-Hailey patients (the most severe ones), experienced a relapse during the study. No serious side effects were reported. Mild transient clear fluid discharge at the site of the injections was reported for 27% of patients.

**Conclusions:**

Botulinic toxin seems to be an effective and safe treatment for Hailey-Hailey and Darier diseases. Nevertheless, it may prove insufficient for the severest of Hailey-Hailey cases and could be considered as supplementary to other conventional treatments. Further studies are required to confirm our results on larger Darier cohorts.

## Background

Darier disease (DD) and Hailey-Hailey disease (HHD) are two rare genetic diseases sharing some clinical (recurrent inflammatory erythematous plaques with a predilection for the skin folds), histopathological (acantholytic dyskeratosis) and genetic (inherited as autosomal-dominant traits, mutations in genes encoding for Ca2+ ATPases, *ATP2A2* for DD and *ATP2C1* for HHD) similarities [1]. DD is mainly defined by warty papules in seborrheic and flexural areas, whereas the cardinal symptoms of HHD are vesicles and erosions in flexural skin [2]. Lesions are intermittent or permanent but acute exacerbations occur, especially during hot seasons, with a possible worsening with sweating. For both diseases, quality of life (QOL) is impaired because of troublesome symptoms such as pain, itchiness and a predilection for cutaneous infections [3–5].

There are currently no curative treatments. Several therapeutic options, like topical agents [6–10], systemic therapies [11–18] and interventional treatments [19–29], are available but HHD and DD are often difficult to control. Botulinum toxin type A (BtxA) may be another option. BtxA has proved effective outside the field of Dermatology. In Dermatology, intracutaneous injections of BtxA have been used to treat focal hyperhidrosis [30–32] and skin conditions such as pachyonychia congenita [33]. BtxA blocks the release of acetylcholine from the sympathetic nerve fibres that stimulates the eccrine sweat glands and causes a localised reduction in sweating. An improvement in skin lesions following BtxA injections has previously been reported in a few patients suffering from both DD and HHD [34–43], but no prospective interventional studies are available. The aim of this study was to evaluate the effect of BtxA injections in the flexural skin of HHD and DD patients.

## Material and methods

This was an open-label, 6-month, interventional pilot study conducted in an expert centre. The study protocol was approved by the Institutional Ethics Committee (*Comité de Protection des Personnes*—Aquitaine, Bordeaux, France) and the French National Agency for Medicines and Health Products Safety, ANSM (*Agence Nationale de Sécurité du Médicament et des produits de santé*). The study was conducted in accordance with the principles of the 1975 Declaration of Helsinki, revised in 1983, and registered under the ClinicalTrials.gov identifier NCT02782702.

### Patients

According to pilot study methodology [44], 30 patients were expected to be enrolled in this study. Eligible patients were aged ≥ 18 years and had a clinically and biopsy-proven diagnosis of DD or HHD. At baseline, patients were required to present at least one lesion located in the large fold areas (inguinal, axillary, mammary and abdominal), ranging from moderately severe to very severe (see section on outcomes). Exclusion criteria were provided as Additional file 1. Written consent was obtained from all patients.

### Intervention

Each patient was scheduled to attend four visits. At the first visit, each patient received intradermal BtxA injections (Botox^R^) on selected areas presenting moderate to very severe lesions (see section on outcomes). These injections were performed under local anaesthesia (lidocaine prilocaine 5% cream, applied one hour earlier). Not more than one BtxA (200-unit) vial was used per patient. The BtxA vial was diluted with 8 mL of sterile, preservative-free physiological saline solution (final concentration: 2.5 units/0.1 ml). The areas to be injected were divided into a grid of 1 to 2 cm. Using a 30-gauge needle, 2 mL (50 units) were then administered intradermally, divided into a constant volume of 0.1 mL injected into the centre of each grid, resulting in approximately 20 intradermal injections over the entire treated area. The target concentration was 50 IU per 100 cm^2^. There were 2 follow-up visits (1 and 3 months (M): M1 and M3), and one end-of-study visit (M6). Patients were instructed not to use any concomitant medication throughout the treatment period, except for emollients and antiseptics. In the event of relapse, defined as the occurrence of new skin lesions in the treated areas necessitating one of these prohibited treatments, the patient was excluded from the study (but still followed-up for tolerance outcomes).

### Outcomes

Several parameters were evaluated at each visit: The first parameter was QOL, evaluated by the Dermatology Life Quality Index (DLQI) [45] (a score > 10 is associated with a severe or very severe impact on QOL [46]). We also evaluated psychosocial impairment using HidroQoL [47]; debilitating symptoms (itchiness, cutaneous pain, sweating and odour) using Visual Analogue Scales (VAS) (ranging from 0 (none) to 10 (worst case scenario)); and the number of infectious skin episodes (in the six months before and after the injections). The total surface area (cm^2^) was evaluated by totalling the surface of all selected areas. The severity of the lesions was evaluated on an individual basis using a 5-point photographic scale (no lesion, mild, moderate, severe, very severe), generated by the investigators and five other independent experts (see Additional file 2). Global severity was defined as the average of the severity of each selected area. Clinical improvement was assessed by the treating investigator (AM) as well as a blinded investigator (ID) at M1, M3 and M6, using the IGA score (Improvement Global Assessment) [48], a validated 5-point scale, ranging from 0 to 4 (no improvement or exacerbation of the treated lesions, slight improvement, moderate improvement, significant improvement, complete disappearance of all lesions). In the event of discordance, moderation analysis were carried out. Patients were considered in treatment failure if the treated area did not improve throughout the study (non-responders to BtxA) or if they relapsed (occurrence of new skin lesions in the treated areas warranting the use of a prohibited treatment). Finally, tolerance was assessed during the injections using a VAS quoting pain from 0 to 10 (0: no pain, 10: maximal pain), and throughout the study period by reporting side effects. Patient satisfaction was recorded at the end-of-study visit according to a 4-point scale (very satisfied, satisfied, somewhat satisfied, dissatisfied).

### Statistical considerations

A descriptive analysis was performed at each evaluation time (baseline, M1, M3 and M6) for each assessment criterion (mean, standard deviation, minimum–maximum for continuous variables; proportion for categorical variables). Before/after comparisons (each evaluation time *vs*. baseline, no comparison between the different follow-up evaluation times) were then performed using a Wilcoxon test for paired values. Disease severity at baseline (defined by the total surface area and the global severity) was compared between relapsing and non-relapsing patients using a Mann–Whitney test. For all tests, a *p* value of < 0.05 was considered statistically significant. All statistical tests were performed with STATA software.

## Results

A total of 30 patients were enrolled in the study. Their characteristics are reported in Table 1. Most of the patients were suffering from HHD (n = 26, 87%) with an equal percentage of men and women, over 55 years old on average. Mean disease duration exceeded 25 years and all patients had received previous therapies.

**Table 1:** Baseline characteristics of the 30 patients enrolled in the study

	HHD & DD—n (%): 30 (100)	HHD—n (%): 26 (86.7)	DD—n (%): 4 (13.3)
**Patients’ characteristics**			
Age—Mean (± SD) [min–max]	55.8 (± 15.5) [21.6–77.6]	56.2 (± 16) [21.6–77.6]	54.1 (± 13.6) [42–68.5]
Sex Ratio (F/M)	1	1	1
Family history of disease—n (%)	24 (80)	21 (80.8)	3 (75)
Age at first signs (Y)—Mean (± SD) [min—max]	28.9 (± 13.9) [8–72]	29.5 (± 14) [8–72]	24.8 (± 14.5) [13–44]
Age at diagnosis (Y)—Mean (± SD) [min—max]	41 (± 17.2) [14–73]	41.8 (± 17.1) [18–73]	35.3 (± 19.6) [14–58]
Duration of the disease (Y)—Mean (± SD) [min—max]	27.1 (± 16.5) [0.6–59.5]	26.7 (± 17.7) [0.6–59.5]	29.3 (± 4.3) [24.5–34.8]
**Previous treatments**			
*Topical treatments—n (%)*	*30 (100)*	*26 (100)*	*4 (100)*
Antiseptics, emollients, antibiotics	30 (100)	26 (100)	4 (100)
Steroids	30 (100)	26 (100)	4 (100)
Tacrolimus	7 (23)	7 (27)	0 (0)
*Systemic treatments—n (%)*	*18 (60)*	*15 (58)*	*3 (75)*
Antibiotics	17 (57)	15 (58)	2 (50)
Retinoids	3 (10)	1 (4)	2 (50)
Steroids	3 (10)	3 (10)	0 (0)
Antihistamines	2 (7)	2 (7)	0 (0)
*Interventional treatments—n (%)*	*13 (43)*	*9 (35)*	*4 (100)*
Botulinum toxin	6 (20)	5 (19)	1 (25)
CO_2_ laser (laser ablation)	7 (23)	5 (19)	2 (50)
Surgical dermabrasion	1 (3)	1 (3)	0 (0)
Photodynamic therapy	8 (27)	5 (19)	3 (75)
**Disease characteristics**			
*Characteristics of the selected areas*			
Number—Mean (± SD) [min—max]	3 (± 1.2) [1–7]	3 (± 1.3) [1–7]	2.8 (± 1) [2–4]
Location—n (%)			
Axillary fold (uni- or bilateral)	23 (77)	20 (77)	3 (75)
Inguinal fold (uni- or bilateral)	20 (67)	17 (65)	3 (75)
Mammary fold (uni- or bilateral)	7 (23)	5 (19)	2 (50)
Abdominal apron	3 (10)	2 (8)	1 (25)
*Disease severity*—Mean (± SD) [min—max]			
Total surface area (cm^2^)	271.7 (± 208.2) [43–995]	271 (± 220.6) [43–995]	276.3 (± 115.3) [120–398]
Global severity (score/4)	2.89 (± 0.68) [2–4]	2.86 (± 0.69) [2–4]	3.04 (± 0.67) [2.5–4]
*Quality of life and psycho-social impairment*			
DLQI (score/30)—Mean (± SD) [min–max]	13 (± 6.84) [1–26]	13.35 (± 6.4) [1–26]	10.75 (± 10.14) [1–20]
DLQI > 10—n (%)	20 (66.7)	18 (69)	2 (50)
HidroQoL (score/36)—Mean (± SD) [min—max]	18.3 (± 7.8) [5–35]	18.62 (± 7.86) [5–35]	16.25 (± 8.14) [7–26]
*Functional symptoms*—Mean (± SD) [min–max]			
Itchiness (VAS/10)	4.3 (± 2.71) [0–10]	4.05 (± 2.79) [0–10]	5.88 (± 1.44) [5–8]
Cutaneous pain (VAS/10)	4.63 (± 2.86) [0–9]	4.8 (± 2.69) [0–9]	3.53 (± 4.07) [0–7.1]
Sweating (VAS/10)	5.8 (± 2.65) [0–10]	5.64 (± 2.78) [0–10]	6.88 (± 1.31) [5–8]
Odour (VAS/10)	4.79 (± 3.05) [0–10]	4.46 (± 3.09) [0–10]	6.93 (± 1.72) [5–8.7]
*Skin infections during the 6 months preceding the injections*			
Number of episodes—Mean (± SD) [min–max]	5.69 (± 8.16) [0–40]	5.44 (± 8.33) [0–40]	7.25 (± 7.89) [0–18]

Legend: D: Darier disease; DLQI: Dermatology Life Quality Index; F: Female; HHD: Hailey-Hailey disease; HidroQoL: Hyperhidrosis Quality of life index; M: Male; [min—max]: [minimum—maximum]; n: number of patients; %: percentage; SD: Standard Deviation; VAS: Visual Analogue Scale; Y: year

**Table 2:** a and b: Effects of Botulinum toxin type A on the 30 patients enrolled in the study (2a), with details given separately for HHD and DD (2b)

	M1		M3		M6	
**Dropped-out patients—n (%)**	9 (30)		9 (30)		11 (36.7)	
Relapsing patients between [study period]: n (%)	[M0–M1]: 9 (30)		[M1–M3]: 0 (0)		[M3–M6]: 2 (6.7)	
**Ongoing patients—n (%)**	**21 (70)**		**21 (70)**		**19 (63.3)**	
Improvement Global Assessment (IGA)—n (%)						
IGA0	1 (3.3)		2 (6.7)		4 (13.3)	
IGA1	3 (10)		1 (3.3)		2 (6.7)	
IGA2	2 (6.7)		4 (13.3)		0 (0)	
IGA3	9 (30)		7 (23.3)		6 (20)	
IGA4	6 (20)		7 (23.3)		7 (23.3)	
Effectiveness parameters—mean score (± SD) & *p* value^a^						
*Disease severity*						
Total surface area (cm^2^)	161.5 (± 310.2)	***p = 0.0002***	106.2 (± 162.9)	***p < 0.0001***	71.4 (± 126.6)	***p < 0.0001***
Global severity (score/4)	2.17 (± 1.14)	***p = 0.004***	2.20 (± 1.24)	*p* = 0.10	1.99 (± 1.09)	***p = 0.02***
*Quality of life and psycho-social impairment*						
DLQI (score/30)	8.73 (± 9.48)	***p = 0.006***	5.14 (± 6.27)	***p < 0.0001***	4.5 (± 5.93)	***p = 0.0001***
HidroQoL (score/36)	12.47 (± 10.77)	***p = 0.001***	8.23 (± 8.55)	***p < 0.0001***	8.42 (± 6.77)	***p = 0.0008***
*Functional symptoms*						
Itchiness (VAS/10)	1.96 (± 2.82)	***p = 0.0002***	1.82 (± 2.31)	***p = 0.003***	1.61 (± 2.4)	***p = 0.001***
Cutaneous pain (VAS/10)	1.82 (± 2.41)	***p < 0.0001***	1.64 (± 2.32)	***p = 0.0004***	1.85 (± 2.8)	***p = 0.001***
Sweating (VAS/10)	1.92 (± 2.1)	***p < 0.0001***	2.38 (± 2.33)	***p = 0.0006***	2.92 (± 2.05)	***p = 0.0005***
Odour (VAS/10)	1.2 (± 2.21)	***p < 0.0001***	1.89 (± 2.54)	***p = 0.002***	1.97 (± 3.27)	***p = 0.005***
*Skin infections during the 6 months following the injections*						
Number of skin infections episodes (n)	_	_	_	_	0.37 (± 1.21)	**p = 0.002**

	HHD			DD		
	M1	M3	M6	M1	M3	M6
**Dropped-out patients—n (%)**	**9 (30)**	**9 (30)**	**11 (37)**	**0 (0)**	**0 (0)**	**0 (0)**
Relapsing patients between [study period]: n (%)	[M0–M1]: 9 (30)	[M1–M3]: 0 (0)	[M3–M6]: 2 (6.7)	[M0–M1]: 0 (0)	[M1–M3]: 0 (0)	[M3–M6]: 0 (0)
**Ongoing patients—n (%)**	**17 (57)**	**17 (57)**	**15 (50)**	**4 (13.3)**	**4 (13.3)**	**4 (13.3)**
Improvement Global Assessment (IGA)—n (%)						
IGA0	1 (3.3)	1 (3.3)	3 (10)	0 (0)	1 (3.3)	1 (3.3)
IGA1	0 (0)	0 (0)	1 (3.3)	3 (10)	1 (3.3)	1 (3.3)
IGA2	2 (6.7)	3 (10.1)	0 (0)	0 (0)	1 (3.3)	0 (0)
IGA3	8 (26.8)	6 (20.1)	5 (16.7)	1 (3.3)	1 (3.3)	1 (3.3)
IGA4	6 (20.1)	7 (23.5)	6 (20)	0 (0)	0 n	1 (3.3)
Effectiveness parameters—mean score (+ SD) & *p *value^a^						
*Disease severity*						
Total surface area (cm^2^)	143.4 (± 321.9)	72.7 (± 136.4)	40.7 (± 53.6)	279.5 (± 212.9)	256.9 (± 208.3)	186.5 (± 245.6)
*(p value)*	***(p < 0.0001)***	***(p < 0.0001)***	***(p = 0.0003)***			
Global severity (score/4)	2.28 (± 1.13)	2.38 (± 1.19)	2.08 (± 1.14)	1.5 (± 1.14)	1.38 (± 1.25)	1.67 (± 0.91)
*(p value)*	*(p* = *0.14)*	*(p* = *0.06)*	*(p* = *0.37)*			
*Quality of life and psycho-social impairment:*						
DLQI (score/30)	8.69 (± 9.73)	4.33 (± 5.4)	4.19 (+ 4.82)	9 (± 8.91)	8.75 (± 9.39)	5.75 (± 10.21)
*(p value)*	***(P = 0.009)***	***(p < 0.0001)***	***(p = 0.0003)***			
HidroQoL (score/36)	12.46 (± 10.71)	7.44 (± 7.86)	9.06 (± 7.03)	12.5 (± 12.87)	11.75 (± 11.87)	5 (± 4.58)
*(p value)*	***(p = 0.003)***	***(p < 0.0001)***	***(p = 0.003)***			
*Functional symptoms*						
Itchiness (VAS/10)	1.82 (± 2.87)	1.44 (± 2.16)	1.5 (± 2.58)	2.8 (± 2.67)	3.5 (± 2.52)	2.05 (± 1.67)
*(p value)*	***(p = 0.002)***	***(p = 0.008)***	***(p = 0.01)***			
Cutaneous pain (VAS/10)	1.87 (± 2.51)	1.56 (± 2.39)	1.81 (± 2.88)	1.5 (± 1.91)	2 (± 2.31)	1.98 (± 2.88)
*(p value)*	***(p < 0.0001)***	***(p = 0.0009)***	***(p = 0.001)***			
Sweating (VAS/10)	1.75 (± 1.92)	2.18 (± 1.94)	2.56 (± 1.86)	3 (± 3.16)	3.25 (± 3.95)	4.35 (± 2.4)
*(p value)*	***(p < 0.0001)***	***(p = 0.005)***	***(p = 0.003)***			
Odour (VAS/10)	1.15 (± 2.29)	1.53 (± 2.45)	1.59 (± 3.14)	1.5 (± 1.91)	3.5 (± 2.55)	3.45 (± 3.87)
*(p value)*	***(p < 0.0001)***	***(p = 0.01)***	***(p = 0.03)***			
*Skin infections during the 6 months following the injections*						
Number of skin infections episodes (n)	_	_	0.33 (± 1.29)	_		0.5 (± 1)
*(p value)*			***(p = 0.01)***			

^a^Wilcoxon test for paired values (comparison between each evaluation points versus baseline); *p *value considered statistically significant when < 0.05 (bold print)

Legend: DLQI: Dermatology Life Quality Index; HidroQoL: Hyperhidrosis Quality of Life Index; IGA: Investigator Global Assessment; M1: 1st month after BtxA injections; M3: 3rd month after BtxA injections, M6: 6th month after BtxA injections; n: number of patients; %: percentage; SD: Standard deviation; VAS: Visual Analogue Scale

The mean (± SD) number of selected areas per patient at baseline [min–max] was 3 (± 1.2) [1–7], with the axillary fold being the main focus (77% of patients). The mean (± SD) global severity score [min—max] was 2.9 (± 0.81) [2–4], corresponding to 17% moderate, 43% severe and 40% very severe lesions. The mean (± SD) total treated surface area per patient [min—max] was 272 cm^2^ (± 208.2) [43—995], resulting in a mean (± SD) dose of BtxA [min—max] of 125 IU (± 61.6) [20–200], with respect to the target concentration.

QOL was impaired for all patients, with a mean (± SD) DLQI score [min–max] of 13 (± 6.84) [1–26], and 20 (66.7%) patients reporting a severe to very severe impact on their QOL. Severe psychosocial impairment was also noted with a mean (± SD) HidroQoL score [min–max] of 18.3 (± 7.8) [5–35]. All (100%) patients reported debilitating functional symptoms, predominantly sweating (mean VAS (± SD) score [min–max] of 5.8 (± 2.65) [0–10]).

**Fig. 1:**
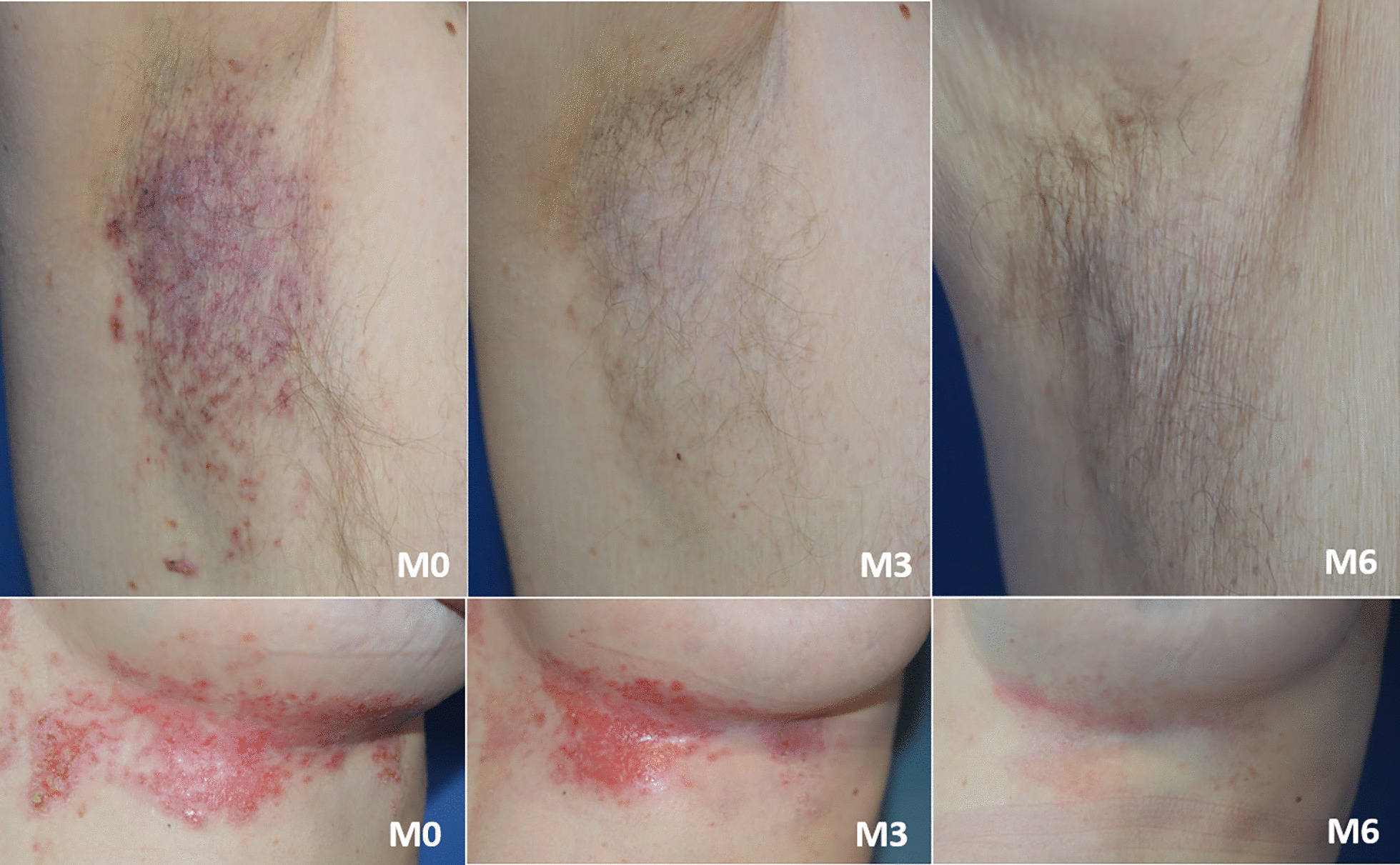
Right axillary fold (top) and left mammary fold (bottom) lesions in a 68 year-old man presenting Hailey-Hailey disease and in a 42 year-old woman presenting Darier disease, both and treated by botulinum toxin: clinical aspect at M0, M3 and M6

The effects of BtxA are presented in Tables 2a and b. A total of 20 (66.7%), 19 (63.2%) and 15 (50%) patients improved (IGA > 0), with a complete disappearance of the lesions in 6 (20%), 7 (23.3%) and 7 (23.3%) patients at M1, M3 and M6, respectively (Fig. 1). No patient was classed as a BtxA non-responder, but 11 (36.7%) patients (11 HHD) experienced a relapse during the study: 9 (30%), 0 (0%) and 2 (6.7%) patients at M1, M3 and M6, respectively. The condition of these HHD relapsing patients was more severe at baseline compared to non-relapsing patients, with a mean (± SD) global severity score [min–max] of 3.21 (± 0.65) [2.5–4] vs*.* 2.7 (± 0.64) [2–4], *p* = 0.05. Relapses were recorded in all locations and occurred in areas with very severe (45.5%), severe (45.5%) or moderate (9%) baseline lesions. The total surface area at baseline did not differ statistically between relapsing and non-relapsing patients (mean (± SD) total surface area [min–max] of 323 cm^2^ (± 294.8) [44–995] vs. 242.1 cm^2^ (± 137.9) [43–553], *p* = 0.81).

**Fig. 2:**
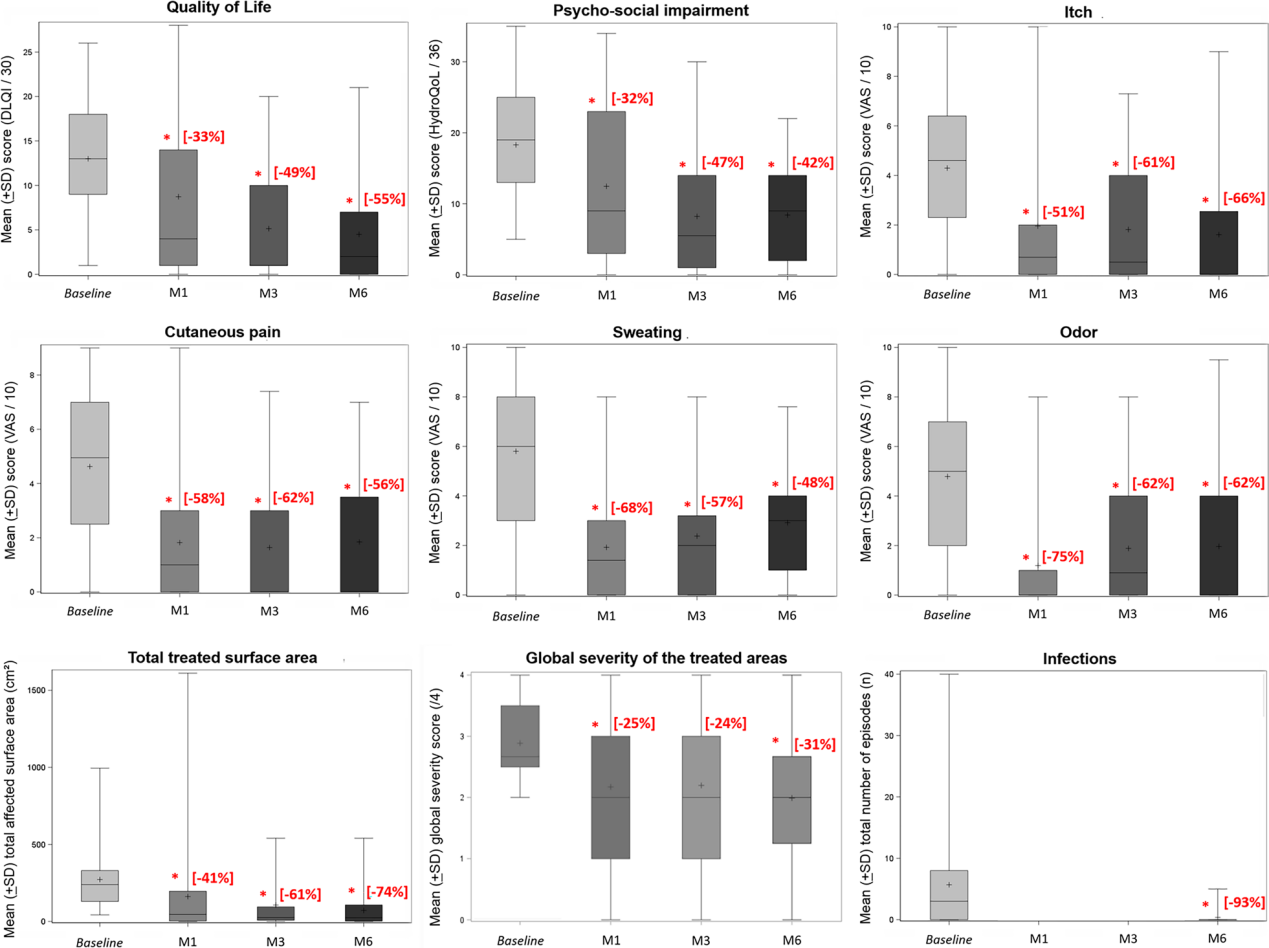
Effects of botulinum toxin and the different outcomes (quality of life, psychosocial impairment, itchiness, cutaneous pain, sweating, odour, infections, total treated surface area and global severity of treated areas): [percentage variation from the mean score at baseline]. * statistically significant *p *value (≤ 0.05)

When considering the HHD and DD non-relapsing patients (Table 2a, Fig. 2), all study parameters were significantly improved by BtxA, with a reduction in values from 24 to 93%, compared to baseline. This reduction was observed from M1 onwards, and persisted over time, remaining significantly lower at M6 compared to baseline, for all values. Only sweating and odours tended to rise once again after M1. The results were similar when detailing per disease (Table 2b), even though the DD cohort is too small to perform statistical analysis separately. When focusing on HHD patients, the mean (± SD) total surface area per patient was reduced by 85% (*p* = 0.0003). In the same way, QOL improved substantially, with the DLQI score that reduced by more than two-thirds (*p* = 0.0003) at M6. Lastly, skin infections were virtually non-existent during the study period (0.33 (± 1.29), *p* = 0.01).

BtxA injections were fairly well tolerated by all patients, with a mean (± SD) pain score during the injections [min–max] of 3.58/10 (± 2.3) [0–8]. Seven patients (23%) experienced transient and very slight side effects during the injections (bleeding (16.5%), tingling sensations (6.5%)). Eight patients (27%) reported a mild, debilitating, clear fluid discharge from the treated areas starting within the first few days of injections and persisting for less than one month. No systemic side effects attributable to the BtxA injections were reported during patient follow-up. Finally, 12 patients (40%) declared that they were very satisfied with BtxA injections (9 (30%) satisfied/2 (6.7%) somewhat satisfied/7 (23.3%) dissatisfied, including 5 relapsing patients, whose dissatisfaction was related to discontinuation of their usual treatments, underlying insufficient global efficacy and relapse).

## Discussion

This is the first prospective study aiming to demonstrate the effectiveness and safety of BtxA injections in the management of DD and HHD. Except for the most severe HHD patients, BtxA is effective within the first month taking all study parameters into account including QOL, and continues to be effective during the 6-month follow-up period.

There are some limitations to our study: the small number of patients (especially with DD), thereby precluding some statistical analysis because of a lack of statistical power the pilot design preventing a control group (placebo group or intra-individual comparison between treated and untreated/placebo treated sides), the highly variable spontaneous course (especially of HHD) with exacerbations and remissions and the absence of information about late relapses (follow-up limited to 6 months). Given the lack of validated scores, we generated a 5-point photographic severity scale. No psychometric validation of this scale was performed in this study. Nevertheless, the fact that clinical severity (rated with this scale) and the other study parameters are moving in the same direction is indicative of the suitability of this tool.

Our results are difficult to compare with those of published small-scale retrospective case series or some clinical cases. In DD, BtxA was used in only three patients in whom a reduction in skin lesions in the treated areas was reported without any objective quantification [49–51]. In HHD, BtxA proved effective in 27 patients (small-scale retrospective case series including no more than eight patients [43] or clinical cases [34–42]). Full or partial remissions were reported using variable dosages of BtxA. Maintenance sessions with variable intervals were initiated for some patients because of relapses occurring after 4–17 months.

Our study confirms the constant, rapid and persistent effectiveness of BtxA with specific information on the improved parameters over a 6-month period. Our study is also the first to demonstrate an impact on QOL, deemed to be the most important parameter when assessing the impact of a new intervention [52].

An important aspect of our study is the characterisation of relapsing HHD patients, who mostly corresponded to the most severe cases. These relapses were mostly early-onset and may have been influenced by the discontinuation of previous therapies not permitted during the study (i.e. local steroids). For those patients, the dosage of BtxA may also have been too low. To our knowledge, no data are available regarding the management of HHD and DD with such higher dosages, but BtxA safety data in other diseases such as spasticity suggest that a maximum dose of 1000 IU per injection or 30 IU/kg per patient per injection (whichever is lowest) may be used [53]. BtxA may also be used as a combined treatment. Surgical ablation which can cover much larger affected areas remains the treatment of choice in intertriginous HHD and DD diseases.

Similar to the literature, few slight side effects such as tingling and bleeding during the injections were reported in our study. Transient fluid discharge has not been previously reported. After questioning patients, this fluid discharge could be indicative of increased oozing from abraded skin.

This study extends the therapeutic use of BtxA beyond aesthetic medicine [54] and enhances our knowledge of the mechanism of BtxA. The fact that predilection for recurrent sweating and odours after M1 did not correlate with diminished efficacy may suggest that BtxA not only reduced sweating, but may have other biological effects in non-neuronal cells such as an anti-inflammatory effect [33].

## Conclusion

In conclusion, BtxA seems to be a safe and effective treatment in the management of intertriginous HHD and DD, thereby expanding the treatment portfolio available. Nevertheless, it may prove inadequate for the severest of HHD cases and has to be used at higher dosages or in combination with other conventional treatments. Further studies are required to confirm our results on larger DD cohorts.

## Supplementary Information

**Additional file 1.** Exclusion criteria.

**Additional file 2.** The 5-points photographic scale to evaluate HHD and DD clinical severity.

## Data Availability

The datasets generated, used and analysed during the current study are available from the corresponding author on reasonable request.

## Abbreviations

ANSM: Agence Nationale de Sécurité du Médicament et des produits de santé
BtxA: Botulinum toxin type A
DD: Darier disease
DLQI: Dermatology Life Quality Index
HHD: Hailey-Hailey disease
IGA: Improvement Global Assessment
QOL: Quality of life
VAS: Visual Analogue Scales
