# Supplementary material for: Botulinum toxin injections as an effective treatment for patients with intertriginous Hailey-Hailey or Darier disease: an open-label 6-month pilot interventional study
Source: Orphanet J Rare Dis. 2021 Feb 18;16:93. doi: 10.1186/s13023-021-01710-x (PMC7893874; doi:10.1186/s13023-021-01710-x)
Supplement: Supplementary file 1 — Additional file 1. Exclusion criteria. [file 13023_2021_1710_MOESM1_ESM.docx]

**SUPPLEMENTARY DATA** : **Exclusion criteria**

Exclusion criteria were as follows: patient lost to follow-up; known hypersensitivity to BtxA or its excipients, myastheny, swallowing difficulties, medical history of dysphagia or aspiration pneumonia; pregnancy or breastfeeding; mentally/physically incapable of completing the questionnaires, guardianship patients; cutaneous infections at the first visit; enrolment in another study in the last two months; application of topical agents (except emollients or antiseptics) to the injection site in the last seven days; botulinum toxin injections, dynamic phototherapy or laser therapy performed in the last six months or systemic aminoside treatment in the last fortnight.
