# Supplementary material for: Botulinum toxin injections as an effective treatment for patients with intertriginous Hailey-Hailey or Darier disease: an open-label 6-month pilot interventional study
Source: Orphanet J Rare Dis. 2021 Feb 18;16:93. doi: 10.1186/s13023-021-01710-x (PMC7893874; doi:10.1186/s13023-021-01710-x)
Supplement: Supplementary file 2 — Additional file 2. The 5-points photographic scale to evaluate HHD and DD clinical severity. [file 13023_2021_1710_MOESM2_ESM.docx]

**SUPPLEMENTARY DATA** : **The 5-points photographic scale to evaluate HHD and DD clinical severity**

| **Severity of affected area** | **HHD** | | **DD** | |
| --- | --- | --- | --- | --- |
| **0: None** | No cutaneous lesion | | | |
| **1: Mild** | Slightly inflammatory basis  (< 30cm²) | 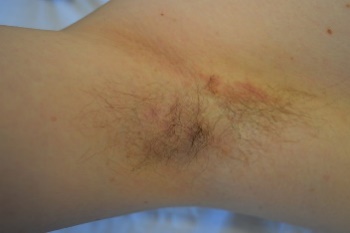 | Sparse and slightly inflammatory papules (that may be slightly hyperkeratotic) | 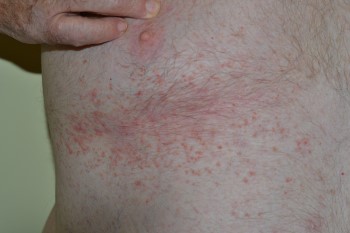 |
| **2: Moderate** | Small erosions on a moderate (light red color) inflammatory basis  (< 30cm²) | 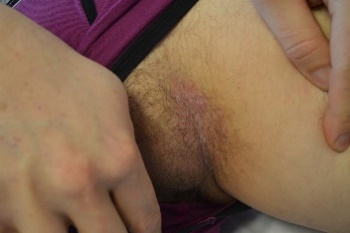 | Numerous hyperkeratotic papules with moderate inflammation  (light red color) | 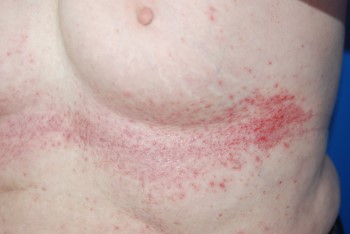 |
| **3: Severe** | Non confluent erosions on a severe (bright red color) inflammatory basis  (< 30cm²)  Oozing, moderate maceration | 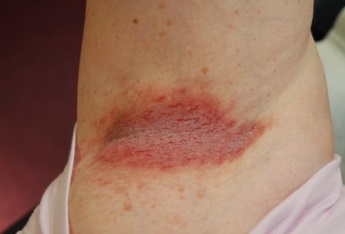 | Confluent (< 30 cm²) hyperkeratotic papules with severe inflammation (bright red color) | 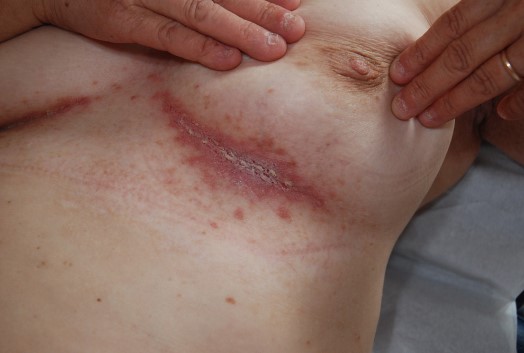 |
| **4: Very severe** | Confluents erosions on a very severe (bright red or purplished color) inflammatory basis (> 30cm²)  Oozing, moderate maceration | 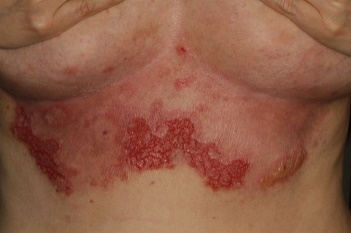 | Confluent and extensive (> 30 cm²) hyperkeratotic papules with very severe inflammation (bright red or purplished color) | 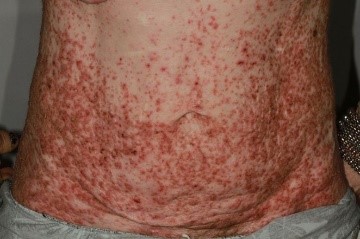 |
